# Addiction to medical websites post COVID-19 pandemic: a predictor of illness anxiety disorder among Arabian youth

**DOI:** 10.1007/s44202-023-00067-5

**Published:** 2023-03-20

**Authors:** Mohamed S. Hamid, Eid Abo Hamza, Rita M. Rivera, Denise Carballea, Nagwa Ibrahim A. Mohamed

**Affiliations:** 1 Present Address: Department of Mental Health, College of Education, Ain Shams University, Cairo, Egypt; 2 College of Education, Humanities & Social Sciences, Al Ain University, Al Ain, UAE; 3 Faculty of Education, Tanta University, Tanta, Egypt; 4 Albizu University-Miami, Doral, USA

**Keywords:** Illness anxiety disorder, Hypochondriasis, COVID-19, Internet addiction

## Abstract

**Background:**

The coronavirus (COVID-19) pandemic has significantly impacted every region of the world. During these unprecedented times, college students have been experiencing severe mental health issues concerning excessive internet usage. On average, 42.9% of students in Egypt utilized the internet (Anwar et al. in J Public Health 30:1753–1762, 2022). Arabs quickly diagnose themselves online using medical websites. The issue is exacerbated by drugs bought without a prescription (Alghadeer et al. in Saudi Pharm J 26:719–724, 2018).

**Methods:**

This study examined he relationship between addiction to medical websites and behaviors related to Illness Anxiety Disorder among a population of Arabic university students. A sample consisting of N = 368 youths was examined.

**Results:**

Bivariate linear regression, Z score, R2, t-test, ANOVA, mean, and standard deviation were used for statistical analysis. The findings of the study revealed a linear equation that predicts illness anxiety in adolescents. The correlation between medical website addiction and hypochondria was found to be 0.69. Furthermore, male participants were more addicted to medical websites than female participants.

**Conclusions:**

Findings supported the notion that addiction to medical websites significantly predicts hypochondria.

## Introduction

Exploring online content over the internet has been the primary source of information flow in today’s world, especially among youngsters during the COVID-19 pandemic. Online sources of information are at the fingertips, and most people use this information in their daily routine. Since it is an essential source of health information, internet usage is immensely boosting in every corner of the globe. According to Ryen and Eric [30], 89% of the USA relies on the internet to fetch medical-related information and answer their concerned queries. Similarly, the internet users hit 75% in Europe related to web browsing on medical issues [32]. A clinical study found that more or less 70,000 websites are operational to disseminate health information that actively engages and connects more than 50 million individuals around the globe via their nexus [8]. Nevertheless, excessive internet usage involves several disadvantages. Such is the case with internet usage related to medical websites, as it has been associated with depression and anxiety among young people, especially those with pre-existing mental health issues. Accordingly, the misconception of data over the internet may lead to overlapping medical concepts and may have desperate consequences.

A plethora of past studies has unveiled that people with no or less medical background suffer from more anxieties while exploring excessive internet for medical consultations [30]. Similarly, Chiu [7] investigated internet users’ interactions with an asynchronous online medical consultation website and reported that most sample members researched for reassurances, confirmation, or diagnosis of medical conditions. In similar research, Singal and Kohli [31] claimed that individuals using online medical websites rely on available resources to diagnose themselves. The major issue in self-diagnosing and consultations is the lack of medical background and reliability of medical websites. According to Benigeri and Pluye [5], a mixed flow of information is available, and few people verify the reliability. Due to the reasons mentioned above, such as excessive internet usage (i.e., addiction) and mixed information, health anxiety may increase among many individuals.

Misuse of internet sources for medical consultations occurs throughout the world in different nations, including most Arab countries. Several individuals suffer from poor health awareness due to several reasons. Most Arabs use online sources to diagnose themselves via online medical websites quickly. Furthermore, the situation is worsened when individuals purchase medications without prescriptions, such as antibiotics [1, 26]. Moreover, studies have reported that this improper method of self-diagnosing among internet users further complicates their medical situation and impacts their psychological well-being [35].

Regarding medical websites related to self-diagnosing and treatment, Coronavirus disease (COVID-19) is a significant breakthrough. The novel virus has challenged common conceptions, resulting in an increase in individuals' web searching behavior related to symptoms, prevention, and treatment. There is hardly a person in any community around the world who is not connected in some way with COVID-19 information. These actions are confirmed by the 2020 report of Google search results, recording “coronavirus” as the first trend in “searches” and “news,” which concerns the general public around the world [17]. Various surveys analyzing internet search data confirmed that Egypt was the first African country to record its first case of COVID-19. Following this diagnosis, there has been a massive increase in web searching for COVID-19 symptoms, which started on February 20th of 2020, as reported by [13]. Additionally, research has indicated that the excessive internet usage during the COVID-19 outbreak may have led to higher levels of depression, anxiety, and compulsive behaviors among internet users [13], as well as boosted overall internet addiction [11, 21, 27]. Addictive patterns such as visiting online medical and health websites qualify as forms of behavioral addiction. During these web searches, individuals aim to examine specific medical information, which may be obtained from specialized, complex, and intertwined searches. This pattern of behavior may lead to the emergence or development of several mental disorders, such as obsessive-compulsive disorder (OCD) and paranoia. Additionally, these addictive patterns and compulsions may serve as behavior that sustains irrational and abnormal ideas. For example, individuals may adopt ritualistic behavior to diminish the fear of contracting a medical condition [29].

Apart from several other addictions related to behavior, the excessive use of the internet is recognized as one of the leading addictions among youths and has been associated as part of the criteria for Illness Anxiety Disorder (IAD) in the Diagnostic and Statistical Manual of Mental Disorders (DSM-V). According to the DSM-V, the IAD is characterized by the preoccupation or fear of contracting or having an illness or serious medical condition [2]. Furthermore, individuals with IAD, colloquially known as hypochondriasis, may constantly seek or avoid medical attention and may present mild somatic symptoms related to this preoccupation. People with this diagnosis often suffer from a misunderstanding of signs and physical symptoms. Individuals may remain surrounded by fear and anxiety despite reassurance or medical evidence. The uncertainty lies in whether people who overuse medical websites following the outbreak of the COVID-19 pandemic develop IAD symptomatology. This situation has not been previously experienced, which suggests these symptoms could have arisen during quarantine and/or lockdowns when access to medical services began to be restricted and replaced by telehealth services and modalities. In the current study, we could not find enough evidence that could explain this phenomenon. However, previous clinical studies have confirmed that individuals who have been diagnosed with IAD may have used medical websites, which can exacerbate their anxiety levels [10]. Only a study conducted by Mohammed et al. [25] reported a correlation between excessive browsing of health websites and IAD, but this result emerged from only a single question from the respondents and did not base on a diagnosis according to a scale to measure IAD symptoms.

To the best of our knowledge and research, no prior studies have addressed the following questions. Research that was conducted in response to the aforementioned concerns about many real-world problems with addiction among Arab youth and the usage of medical websites following the COVID-19 pandemic has been noted, this study aims to identify the following interrogations with exhortations. For instance, could the growing addiction to medical websites after the COVID-19 pandemic be a high predictor of developing IAD among college populations? What is the prevalence of addiction to medical websites among college populations? Are there differences between males and females in prevalence rates? Does addiction to browsing medical websites significantly correlate with IAD? For this study, the authors sought to develop a website addiction measure based on the behavioral addiction diagnostic criteria in the DSM-V, and they examined its psychometric characteristics.

## Research questions and hypothesis development

This research aimed to examine addiction to medical websites during the COVID-19 pandemic, especially among the youth of Arabs. The following set of questions served as this study’s research questions:

1. What is the linear equation that predicts the respondents’ hypochondria from their Addiction to Medical Websites, and how does this equation predict the extent of hypochondria?
2. Is there any significant difference between male and female respondents on addiction to medical websites?
3. Is there any significant difference between male and female respondents on hypochondria?

The following hypothesis was developed to evaluate the mentioned research questions.

H_1_: Addiction to Medical Websites will significantly predicts hypochondria

H_0_: Addiction to Medical Websites will not predict hypochondria

H_2_: there will be a significant difference between male and female on Addiction to Medical Websites

H_0_: there is no difference between male and female on Addiction to Medical Websites

H_3_: there will be a significant difference between male and female on hypochondria

H_0_: there is no difference between male and female on hypochondria

## Methodology

### Study design

This study is based on a cross-sectional survey conducted online during the COVID-19 pandemic at the end of 2020 and the beginning of 2021. The questionnaires were designed and sent to the participants via emails, mails, and online survey tools. Participation in the study was voluntary, and no compensation was provided. All participants signed a consent form prior to participation in the study and ethical approval was obtained from the Ain-shams University review board IRB. This is a cohort study of male and female university students. The data collection was completed between the months of January 2021 and March 2021.

### Samples

Participants were recruited through invitations from professors of courses among undergraduate students interested in participating in the research study. Participants were given an overview of the nature of the study through this invitation. The total sample size consisted of 368 university students from Ain-Shams University in Cairo, Egypt. This sample consisted of 25.5% male participants (N = 94) and 74.5% female participants (N = 274). Participants were between the ages of 17 to 31 [Mean (M) = 19.8; Standard Deviation (SD) = 2.3].

### Measures

At the beginning of the survey, demographic information was obtained from the participants, including their gender, age, family background (single child or not), family income, and the level of education of their parents. Furthermore, the participants were required to answer whether they were diagnosed with certain medical disorders such as organic brain disease, mental illness, and cognitive impairment. Two different measures were adopted during this research, as follows.

#### Addiction to medical websites Scale (AMWS)

The addiction to medical websites Scale (AMWS) was developed previously and referred to using the diagnostic criteria in the DSM-V [18]. The AMWS was specially developed to accurately measure the symptoms associated with addiction to browsing medical websites. The AMWS consisted of 20 items divided into three major dimensions. The first dimension (independency, preoccupation and obsession) consisted of 7 items (1–4-7–10-13–16-19). The second dimension (psychosocial and social influences) also comprises 7 items (2–5-8–11-14–17-20). Lastly, the third dimension (excessive use) consisted of 6 items (3–6-9–12-15–18). A five-point Likert scale, ranging from 1–5, was used to rate the scale items in this scale. The scale scores were the following: (1) Strongly disagree; (2) Disagree; (3) Neither agree nor disagree; (4) Agree; (5) Strongly agree. The total score of the AMWS ranges from 20- 100.

#### An Arabic version of the multidimensional inventory of hypochondriacal traits (MIHT)

The Multidimensional Inventory of Hypochondriacal Traits (MIHT) is a self-report measure consisting of 31 items and four dimensions to assess components of health anxiety (i.e., affective, perceptual, behavioral, and cognitive) [22]. The total score ranges from 31–155, and each item is rated on a 5-point Likert scale ranging from 1 (strongly disagree) to 5 (strongly agree). The higher the score on the MIHT scale, the higher the reported health anxiety. According to [22] a user’s score below 94 is normal; between 94–124 is considered high, whereas 125–155 is assumed very high. The mental state of each participant was also assessed using the Arabic version of MIHT tool. Previous research evaluated psychometric characteristics of the MIHT and reported strong validity and reliability in assessing health anxiety [15, 24, 34]. The Arabic version of MIHT translated by [18] was used in the present study due to its applicability on IAD with the population of interest and reported validity and reliability for the Arabic population. Reliability and validity analysis for the Arabic version of MIHT was performed on 300 non-clinical Arabian university students.

With the aid of this tool, four different dimensions were tested using different items. The Affective dimension was assessed using items 21, 6, 11, 7, 29, 23, and 12. Moreover, the perceptual dimension was measured using items 3, 30, 9, 17, 19, 13, 28, 22, and 24. For the Behavioral dimensions, the items consist of 5, 14, 26, 16, 18, 20, 1, and 25. Items 31, 8, 2, 27, 10, 15, and 4 compromised the cognitive dimension. Participants in the survey who reported frequently browsing medical websites focused mainly on COVID-19 were included in the study analysis procedure. A total of 319 (86.7%) participants were included, and a total of 49 who denied frequent browsing websites related to COVID-19 (13.3%) were excluded. The final sample consisted of 319 participants, from which 81 (25.4%) were male, and 238 (74.6%) were female. The age range of the final sample was from 17–31 (M = 19.8; SD = 2.3).

### Data analysis

The online data from all sources were extracted and compiled. Descriptive analysis in the form of mean value and standard deviation was performed. The analyses were performed using Statistical Package for Social Sciences SPSS 26. A bivariate linear regression analysis was conducted to evaluate the prediction of hypochondria from reported addiction to medical websites. An independent-samples *t*-test was conducted to evaluate the differences between male and female participants. Furthermore, Z score, R^2^, ANOVA, and Leven’s test for equality of variance were also conducted to show various correlations, differences, and significance of data. The test was conducted on a 95% confidence level with a significance level of p < 0.05.

## Results

### Demographic information of participants

In total, 368 surveys were administered where 49 participants did not participate entirely or partially. Out of 319 participants, 81 were male, and 238 were females. The participants were classified among various qualitative measurable items, as demonstrated in Table 1. The participants were classified into normal internet users (normally addictive), high (highly addictive), and very high (excessively addictive).

**Table 1:** Demographic characteristics comparison among normally, highly, and excessively addictive participants

		Normally addictive (N = 154)	Highly addictive (N = 53)	Excessively addictive (N = 112)
Gender	Male	39 (25.32%)	15 (28.30%)	27 (24.10%)
	Female	115 (74.67%)	38 (71.69%)	85 (75.89%)
Age group	17–20	65 (42.20%)	24 (45.28%)	15 (13.39%)
	21–25	43 (27.92%)	17 (32.07%)	58 (51.78%)
	26–31	46 (29.87%)	12 (22.64%)	39 (34.82%)
Single child	Yes	21 (13.63%)	9 (16.98%)	41 (36.60%)
	No	133 (86.36%)	44 (83.02%)	71 (63.40%)
Monthly family income	Below 1,000 USD	36 (23.37%)	8 (15.09%)	13 (11.60%)
	1000–3000 USD	45 (29.22%)	11 (20.75%)	17 (15.17%)
	3000–5000 USD	54 (35.06%)	13 (24.52%)	22 (19.64%)
	Above 5000 USD	19 (12.33%)	21 (39.62%)	60 (53.57%)
Parent education status	Primary School	21 (13.63%)	11 (20.75%)	18 (16.07%)
	Middle School	32 (20.77%)	9 (16.98%)	19 (16.96%)
	Higher School	68 (44.15%)	7 (13.20%)	12 (10.71%)
	College/University	30 (19.48%)	26 (49.05%)	63 (56.25%)

The authors used SPSS to categorize the age groups based on their severity of internet addiction; the results revealed that the age range of 21 to 25 years was high, although those between the ages of 26 and 31 were less hooked and those between the ages of 17 and 20 were less excessively addicted. The demographic comparison of three different types of internet users is interesting. Around 35.10% of internet users were classified as excessively addictive, whereas 16.61% were highly addictive, and 48.27% were normally addictive. The majority of male users were found highly addictive, and almost similar results were obtained for female users. An excessively addictive age group was found from the age group of 21–25. No such relationship is found for a single child and monthly family income; however, excessive users were categorized into high-income families. Similarly, no such linear relation is obtained for parent education status; however, the participants whose parents’ education lies under the college or university section were classified among excessive addictive users.

### Relation of addiction to medical websites with hypochondria

Data were analyzed to compute the relation between addiction to medical websites and its possible relation to hypochondria. In this regard, a bivariate linear regression analysis was conducted to evaluate the prediction of developing hypochondriasis due to addiction to medical websites. A significant regression equation was found, as depicted in Eq. 1.

1 $$F\left(1, 317\right)= 288.07, p < .000, \& \; {R}^{2} of 0.476$$

**Fig. 1:**
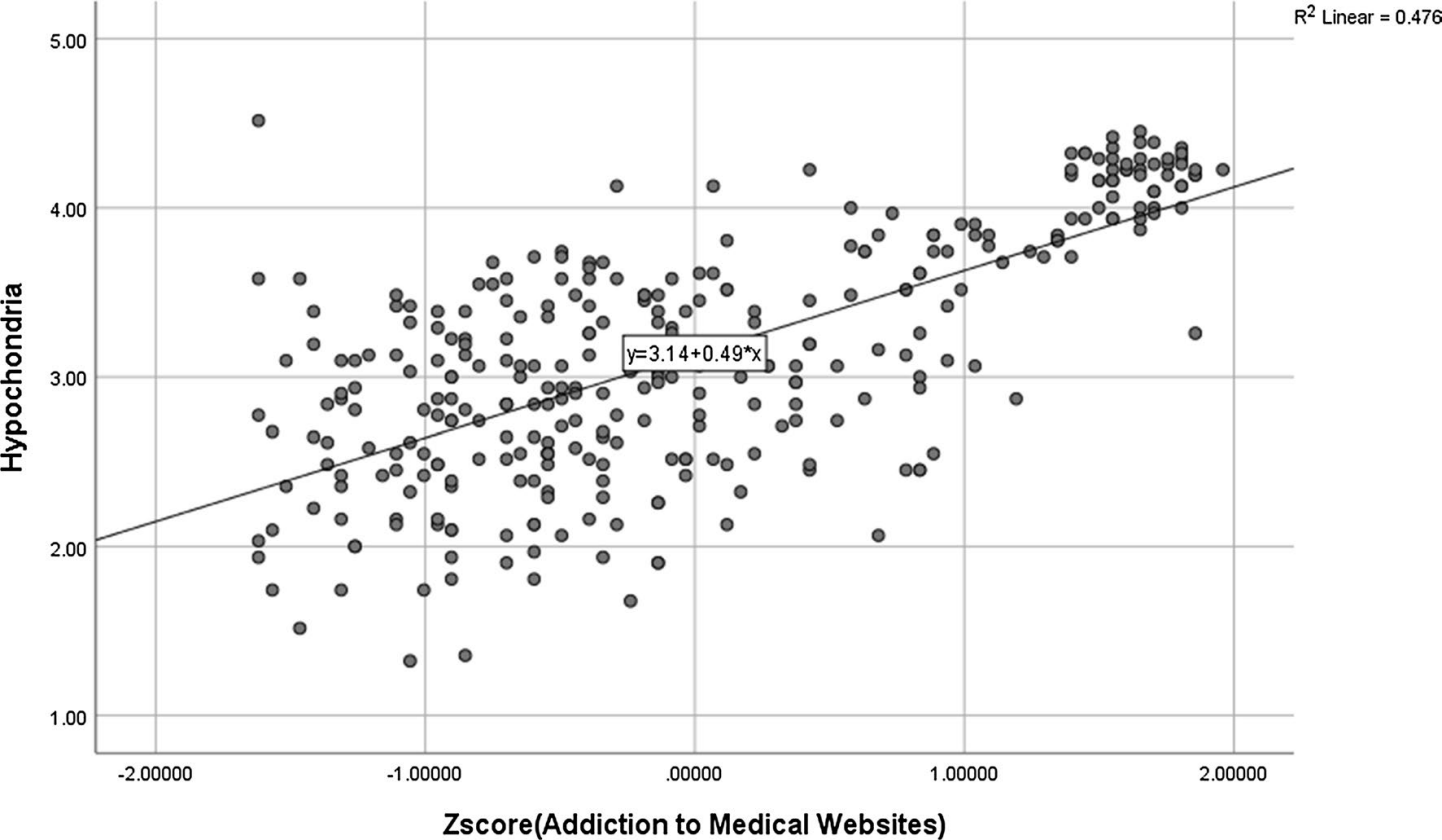
Scatter plot between hypochondria and addiction to medical websites

Figure 1 displays the scatterplot for the two variables, which indicates the two variables were linearly related. This pattern suggests that addiction to medical websites increases overall hypochondriasis. The regression equation for predicting the hypochondria is as shown in Eq. 2.

2 $$Predicted\;Hypochondria=3.14*addiction\;to\;medical\;websites+0.49$$

From the analysis of results, it can be hypothesized that greater addiction to medical websites tended to have a stronger correlation to hypochondria. The correlation between the addiction to medical websites and hypochondria was found as 0.69, as shown in Table 2. Approximately 47.6% of the variance of the hypochondria was accounted for by its linear relationship with the addiction to medical websites (see Table 3). Furthermore, to prove the significance of the relationship between the two variables, i.e., addiction to medical websites and hypochondria, ANOVA analysis was (Table 4) performed. The p-value was tested and found a value of 0.000 (p < 0.05), which shows no significant difference among the two tested variables, which further demonstrates the strong correlation. The 95% confidence interval for the slope is found as 0.437 to 0.551 in Table 5, which does not contain the value of zero. Therefore, suggesting that overall addiction to medical websites is significantly related to overall hypochondria. Overviewing the analysis results indicate that addiction to medical websites significantly predicts hypochondria which proves hypothesis H_1_.

**Table 2:** Correlation between addiction to medical websites and hypochondria

		M_Hypo	Zscore(M_Addiction)
Pearson Correlation	M_Hypo	1.000	0.690
	Z score (M_Addiction)	0.690	1.000
Sig. (1-tailed)	M_Hypo	–	0.000
	Z score (M_Addiction)	0.000	–
N	M_Hypo	319	319
	Z score (M_Addiction)	319	319

**Table 3:** Analysis of R^2^ for the model

Model	R	R^2^	Adjusted R^2^	Std. Error of the estimate
1	0.690^a^	0.476	0.474	0.51898

**Table 4:** Analysis of ANOVA results between addiction to medical websites and hypochondria

Model		Sum of Squares	df	Mean Square	F	Sig. (p)
1	Regression	77.590	1	77.590	288.071	0.000
	Residual	85.382	317	0.269		
	Total	162.972	318

**Table 5:** 95% confidence interval of correlation slope

Model		Unstandardized coefficients		Standardized coefficients	t	Sig	95.0% Confidence interval for B	
		B	Std. error	Beta			Lower bound	Upper bound
1	(Constant)	3.135	0.029		107.903	0.000	3.078	3.193
	Z score (M_Addiction)	0.494	0.029	0.690	16.973	0.000	0.437	0.551

### Relationship of addiction to medical websites with gender differences

An independent-samples *t*-test was conducted to evaluate the hypothesis (H_2_) that there is a significant difference between genders on addiction to medical websites. In this regard, Levene’s Test for Equality of Variances was performed, indicating that the variances for males and females differ significantly from each other (*p* = 0.01 < 0.05). Thus, a *t*-test for *Equal Variances not Assumed* was used. The test was found significant at *t* (129.36) = 7.28, *p* < 0.000, see Table 6. The 95% confidence interval for the means’ difference ranges from 0.64 to 1.12.

**Table 6:** *t*-test for Independent Samples

	Levene's Test for Equality of Variances		*t-*test for Equality of Means						
			*t*	*df*	Sig. (2-tailed)	Mean Difference	Std. Error Difference	95% Confidence Interval of the Difference	
	*F*	Sig						Lower	Upper
Equal variances assumed	6.57	0.01	7.57	317	0.000	0.88	0.12	0.65	1.11
Equal variances not assumed			7.28	129.36	0.000	0.88	0.12	0.64	1.12

**Fig. 2:**
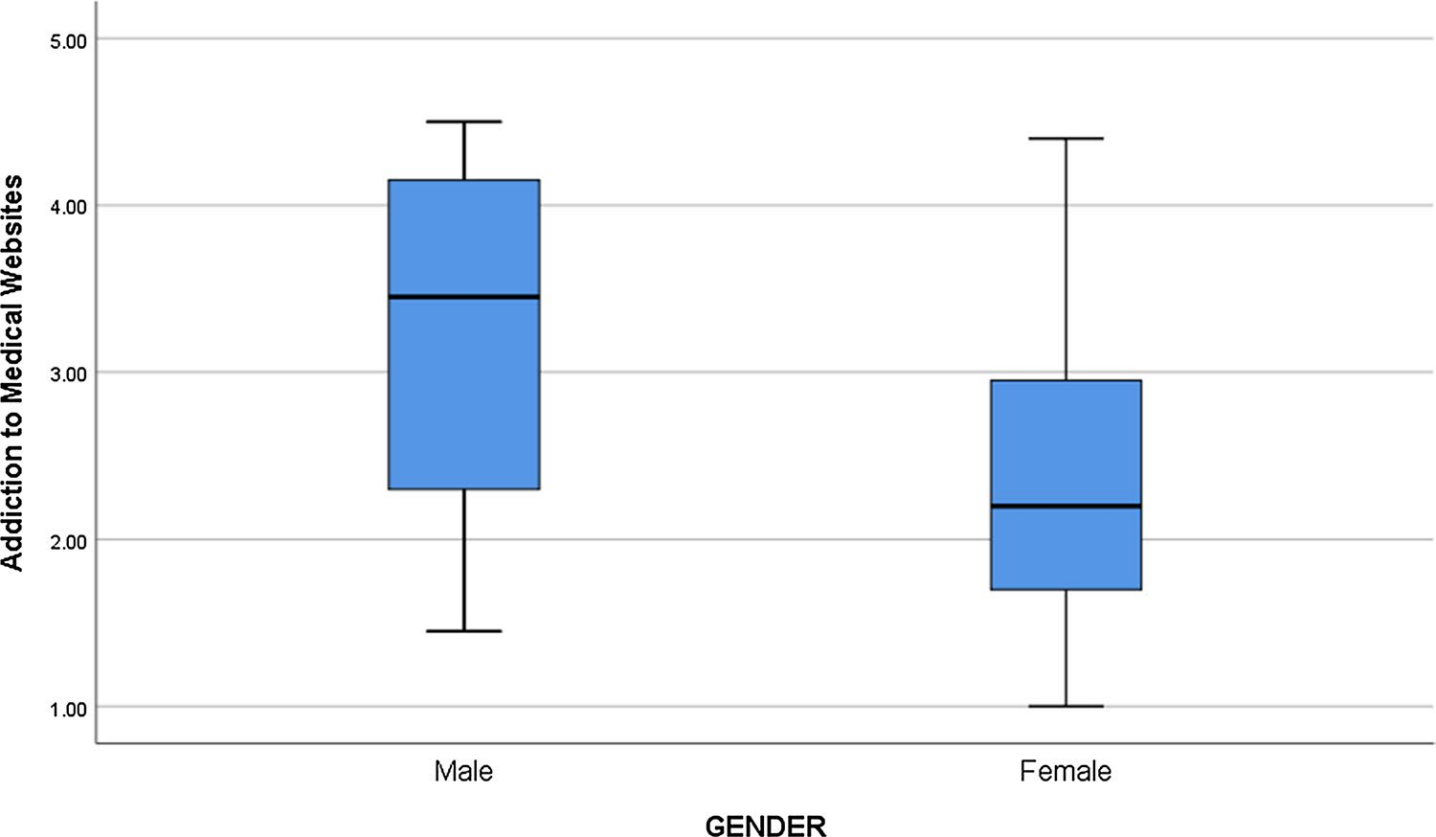
Boxplot of the means and SDs of addiction to medical websites based on gender

Furthermore, for the Male participants, a mean value of 3.24 was found with a (Table 7) standard deviation of 0.96). The results found that, on average, male participants demonstrated higher addiction to medical websites than female respondents, with a mean value of 2.36 and a standard deviation of 0.88. The difference between male and female participants is demonstrated in Fig. 2.

**Table 7:** t-test for Independent Samples

	Levene's Test for Equality of Variances		The *t* test for Equality of Means						
			*t*	*df*	Sig. (2-tailed)	Mean Difference	Std. Error Difference	95% Confidence Interval of the Difference	
	*F*	Sig						Lower	Upper
Equal variances assumed	17.23	0.000	4.42	317	0.000	0.40	0.09	0.22	0.57
Equal variances not assumed			4.00	118.45	00.00	0.40	0.10	0.20	0.59

### Relationship of hypochondria with gender differences

**Fig. 3:**
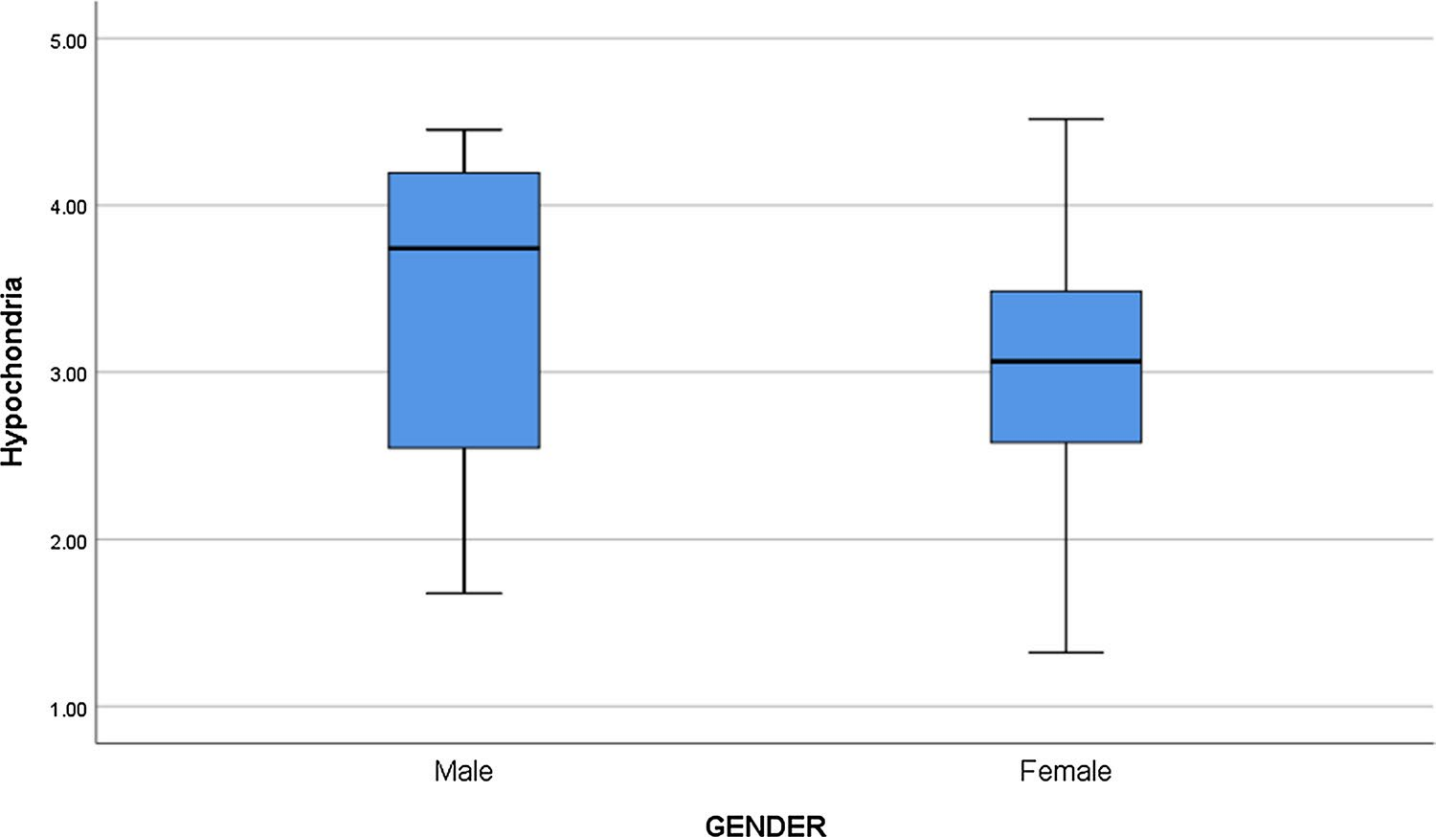
Boxplot of the means and SDs of hypochondria based on gender

An independent-samples *t*-test was conducted to evaluate the hypothesis (H_3_) that there is a significant difference between genders and hypochondria. Levene’s Test for Equality of Variances indicates variances for males and females respondents differ significantly from each other (*p* < 0.000). Thus, a *t*-test for *Equal Variances not Assumed* was used. The test was significant at *t* (118.45) = 4.00, *p* < 0.000. The 95% confidence interval for the means’ difference ranging from 0.20 to 0.59. Furthermore, from the analysis of mean and standard deviation values, we found that the male respondents demonstrated a higher mean of Hypochondria (M = 3.43, SD = 0.80) than female respondents (M = 3.04, SD = 0.66). This difference is also highlighted in Fig. 3.

## Discussion

This study aims to investigate addiction to medical websites during the COVID-19 pandemic period. Various age group participants were involved with gender differences. Three different categories of participants were found, such as normally addictive, highly addictive, and extremely addictive, based on the Arabic version of IAD developed by [19]. The results suggest that the majority of male participants were found under the highly addictive category. In the research sample, there is a higher ratio of females than males; however, the authors do not believe this has a significant effect on the study’s findings because gender was not considered a significant predictor of increased anxiety during the regression analysis. Furthermore, the higher proportion of female samples in this internet-based study is consistent with previous research, which was also conducted on university or college students [14].

The study results show that experienced students have a higher prevalence of internet addiction. According to the findings, the excessively addictive age group within the age range of 21–25 years. This result is in accordance with Lin [21], who also suggests that higher internet addiction is prevalent in those above 20 years of age. Using excessive internet in the post-COVID-19 period leads to inattentiveness among youth and proliferates the chances of developing higher chances of internet addiction to medical websites. In this regard, they are more prone to delay their positive activities. This study discovered that most youth who specifically experience an emotional disturbance may become more prone to medical website browsing [21]. Furthermore, owing to online classes instead of face-to-face, youths spent more time during COVID-19 on the internet than usual [27]. There is another possibility that due to lockdowns, most youths were disconnected from their social circles, which may have also led them to spend unhealthy amounts of time internet browsing. In addition, during these unprecedented times, the youth appear to be very interested in obtaining information about the COVID 19 outbreak, including symptomatology and treatment modalities.

Our study did not find a significant relationship for the participants with a single child and monthly family income; however, excessive users were categorized into high-income families. During the COVID-19 outbreak, the family background played a significant role. The Coronavirus outbreak continues to impact families throughout the world. Generally, interactions between family members is a significant factor of routine life. During this pandemic, individuals were exposed to high-levels of stress due to fear of contracting the novel virus. Higher-income families usually have several engagements with large family social networks; this may be a reason why higher income family backgrounds were among the excessive addiction to medical websites. Similarly, from the analysis of results, no such linear relation is obtained for parent education status; nevertheless, the participants whose parents’ education lies under the college or university section were classified among excessive addictive users. To the best of our knowledge, no previous study is available on Arab youths in post-pandemic circumstances to confirm our findings. However, the possible reason may be that highly educated parents were under higher pressure due to lack of social networks during that time. Parents and caregivers may have also restricted their children’s mobility and forced them to stay in their homes to comply with social distancing regulations.

This study suggests that addiction to medical websites ignites hypochondria among youths. The findings of this research are also in connection with a study by Doherty-Torstrick et al. [10], which supports the same argument. Overall, people suffering from illness anxiety face more fear and functional impairment. These individuals tend to spend more extended periods browsing medical conditions, exposing themselves to triggering health information that may increase their anxiety levels.

Results of this study found that greater addiction to medical websites tended to have a stronger correlation to hypochondria. The correlation between addiction to medical websites and hypochondria was found as 0.69. Furthermore, the relationship between the two variables, i.e., addiction to medical websites and hypochondria, was computed using ANOVA analysis. The p-value was tested and found a value of 0.000 (p < 0.05), which shows no significant difference among the two tested variables, further demonstrating the strong correlation. The findings of this study correlate with findings by Mohammed et al. [25], which reported that the use of internet browsing to seek medical consultations increases anxiety and depression. Patients seeking online consultations about their health assurance may want to avoid physical interaction with doctors and medical experts. In this regard, our study results are in accordance with previous findings [12, 14].

Our study deeply analyzed the relationship among gender with medical websites and hypochondria. According to the findings of this study, the male participants are more addicted than female internet users. Furthermore, a higher risk of hypochondria was found in male participants. There is a significant divergence in the opinion of experts over gender differences. A mixed concept is found from the previous study, and no strong agreement is available on the pages of literature. According to Bahrami and Yousefi [3], females have higher anxiety than males. In contrast Muse et al. [28] reported that gender has no significant relationship with anxiety,however, it depends on their past mental health. Our study’s findings are opposite from the results of Mohammed et al. [25], which suggests females suffer the most from hypochondriasis. However, our study is solely based on the COVID-19 pandemic, which is completely different from previous studies. The possible reason for higher chances of hypochondria in males may be due to the fact that females tend to primarily seek their issues from practitioners physically and sooner than males [23]. In contrast, due to their busy schedule and fear of results, the male tends to avoid physical meets up with health experts, cited by [25]. ‬

The study discovered that Arab youths are more vulnerable to addiction to medical websites, regardless of gender or age group, especially in the aftermath of the COVID-19 pandemic. The study also supports the notion that, despite gender differences, Arab Youths have developed a higher risk of hypochondria. This is regardless of their prior mental health or internet addiction. This study's findings are also consistent with those of Dong et al. [11], who conducted a study in China during COVID-19. Moreover, this study recommends that the youths experiencing illness anxiety avoid excessive internet usage for medical website browsing. This compulsion could be harmful even to those having mild symptoms. These individuals should consider installing filters or blocking software to prevent access to websites that expose individuals health-related information, approaches that have already been successfully applied to other types of pathologic examination [6, 9, 16, 33]. Furthermore, the youth must be more informed in health consciousness, significantly reducing distress, especially from false sources [4, 20].

## Conclusion and future directions

The COVID-19 pandemic has caused significant disruption in the daily lives of all people, particularly young people. People were required to follow social distancing rules such as lockdowns and quarantine periods. This pandemic has increased the internet's prominence, and internet usage has risen dramatically over time. Young people have been searching the internet for information about the COVID-19 pandemic. This activity increased as a result of the limited time available for outdoor activities, which prompted online gaming and internet searching. Excessive internet use may lead to compulsive and addictive browsing of medical websites in young people. As a result, hypochondria appears to be on the rise among young people, particularly in Arab countries.

Through statistical analysis, this study concluded that greater addiction to medical websites had a stronger correlation to hypochondria. The link between addiction to medical websites and hypochondria was discovered to be significant. The ANOVA test also confirms that there is a strong link between medical website addiction and hypochondria. The study also concludes that there is a significant difference between men and women in terms of addiction to medical websites and hypochondria. Males appear to be more susceptible to medical website addiction and hypochondria than females. The study also concludes that, regardless of gender, Arab youths are more disturbed and suffering from severe mental illnesses as a result of the COVID-19 pandemic. This research could be expanded in the future to assess the mental health of heavy internet users. More research is needed to assess the potential solutions to these issues in Arab countries.

## Data Availability

The data that support the findings of this study are available from the corresponding author upon request.
